# Smart Microneedles with Porous Polymer Coatings for pH-Responsive Drug Delivery

**DOI:** 10.3390/polym11111834

**Published:** 2019-11-07

**Authors:** Asad Ullah, Haroon Khan, Hye Jin Choi, Gyu Man Kim

**Affiliations:** School of Mechanical Engineering, Kyungpook National University, Daegu 41566, Korea; sasadullah84@gmail.com (A.U.); mechy_365@yahoo.com (H.K.); hyejin0058@gmail.com (H.J.C.)

**Keywords:** SS microneedles, porous coating, pH-responsive, drug delivery

## Abstract

This work demonstrates a simple approach for coating a porous polymer layer on stainless-steel (SS) microneedles characterized by a pH-responsive formulation for self-regulated drug delivery. For many drug-delivery applications, the release of therapeutic agents in an acidic microenvironment is desirable. Acid-sensitive polymers and hydrogels were extensively explored, but easily prepared polymeric microcarriers that combine acid sensitivity and biodegradability are rare. Here, we describe a simple and robust method of coating a porous polymer layer on SS microneedles (MNs) that release a model drug (lidocaine) in a pH-responsive fashion. It was constructed by packing the model drug and a pH-sensitive component (sodium bicarbonate) into the pores of the polymer layer. When this acid-sensitive formulation was exposed to the acidic microenvironment, the consequent reaction of protons (H^+^) with sodium bicarbonate (NaHCO_3_) yielded CO_2_. This effect generated pressure inside the pores of the coating and ruptured the thin polymer membrane, thereby releasing the encapsulated drug. Scanning electron micrographs showed that the pH-sensitive porous polymer-coated MNs exposed to phosphate-buffered saline (PBS) at pH 7.4 were characterized by closed pores. However, MNs exposed to PBS at pH 5.5 consisted of open pores and the thin membrane burst. The in vitro studies demonstrated the pH sensitivity of the drug release from porous polymer-coated MNs. Negligible release was observed for MNs in receiving media at pH 7.4. In contrast, significant release occurred when the MNs were exposed to acidic conditions (pH 5.5). Additionally, comparable results were obtained for drug release in vitro in porcine skin and in PBS. This revealed that our developed pH-responsive porous polymer-coated MNs could potentially be used for the controlled release of drug formulations in an acidic environment. Moreover, the stimuli-responsive drug carriers will enable on-demand controlled release profiles that may enhance therapeutic effectiveness and reduce systemic toxicity.

## 1. Introduction

Transdermal drug administration has the potential to improve therapeutic delivery, providing an approach that is safer and more convenient than traditional routes, such as oral administration and hypodermic injection. This method offers the opportunity for improved therapeutic effectiveness through sustained and controlled drug release [1]. Compared with more traditional routes, transdermal drug delivery has the potential to provide many practical and clinical advantages [2]. Relative to parenteral injection, transdermal delivery is non-invasive, potentially allowing for rapid, pain-free administration either by minimally trained healthcare providers or through self-administration [3,4]. Transcutaneous delivery systems may minimize the generation of dangerous medical waste and restrain the spread of disease known to occur through needle reuse and needle-based injury [5,6]. Furthermore, dry storage of systems designed for topical application may also provide enhanced drug stability, enabling the transport of environmentally sensitive biological therapeutics without the need for refrigeration. This is a key issue as the requirement of “cold chain” distribution increases costs and inherently limits the availability of therapies throughout the developing world [3]. Transcutaneous therapeutic administration also has the potential to enhance the clinical effectiveness of treatment, by allowing for more efficient delivery (compared with conventional methods) of drugs susceptible to first-pass metabolism in the liver [4]. To this end, a microneedle platform is demonstrated for rapid implantation of controlled-release therapeutic formulation into the cutaneous tissue. When microneedle arrays are applied to the skin, the needles pierce the stratum carneum and epidermis. Penetration of the outer skin layers is followed by the dissolution/diffusion of the payload therapeutic formulation [1]. Recently, microneedle arrays were used as a promising technology for safe and convenient transcutaneous delivery of diverse bioactive materials (including high-molecular-weight hydrophilic biologics) through pain-free mechanical disruption of the stratum corneum [7]. The emergence of smart microcarriers revealed new opportunities for scientists to develop the most efficient anti-disease vehicles with safe and biocompatible profiles [8,9]. Recent developments focused on the fabrication of polymeric devices with multiple biomedical functions to improve the quality of treatment and healthcare management [10]. Microcarriers fabricated from biocompatible and biodegradable polymers through various techniques are widely used for advanced biomedical applications, such as pharmaceutical repositories, tissue engineering scaffolds, wound healing, sensors, and systematic drug delivery [11]. The encapsulated payload is typically released over the course of several months via surface erosion and slow degradation of the polymer [12]. However, for many delivery applications, rapid and selective release of an encapsulated payload by taking advantage of a unique chemical environment is often desirable [13]. Many carrier systems using pH-responsive drug release were developed [14,15]. pH-responsive drug delivery is highly desirable for many biomedical applications. In fact, controlled administration of drugs in a manner that precisely matches physiological needs at targeted sites and at predetermined release rates for predefined periods of time is highly desirable. Different organs, tissues, and cellular compartments have different pH values, rendering the pH value a suitable stimulus for controlled drug release. pH-responsive drug-delivery systems attracted increasing interest as “smart” drug-delivery systems for overcoming the shortcomings of conventional drug formulations. That is, these systems can deliver drugs in a controlled manner at a specific site and time, which results in high therapeutic efficacy [14]. Specifically, for many drug-delivery applications, release of therapeutic agents under mildly acidic conditions (consistent with sites of inflammation, lysosomal compartments, in tumor tissue, or in skin cancers: basal cell carcinoma (BCC), squamous cell carcinoma (SCC), and melanoma) is desirable [16]. In cancer immunotherapy, nanosystems play an essential role in immune cell activation and tumor microenvironment modulation [17]. Acid-sensitive liposomes, micelles, and hydrogels were extensively explored, but easily prepared polymeric materials that combine acid sensitivity and biodegradability are rare [18]. Moreover, most of the smart drug-delivery systems involve many chemical processes and are (in general) time-consuming. The smart microcarriers are prepared through various processes and then administered in the body either by means of hypodermic injection or through combination with microneedles [15,19,20,21]. Therefore, the development of an easily prepared microneedle-based system with the flexibility and biocompatibility of polymer materials, and characterized by a payload release rate that is sensitive to physiologically relevant acidic conditions is considered essential.

**Figure 1:**
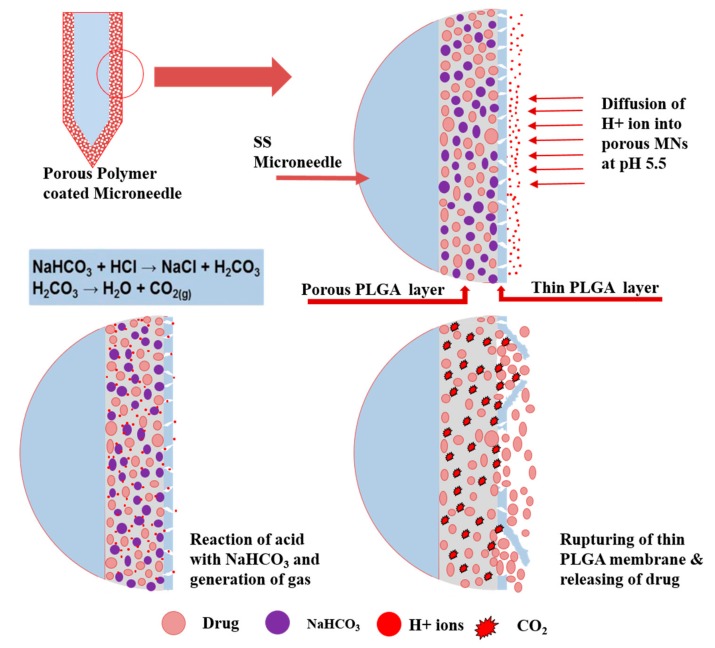
Schematic illustration of the pH-responsive release system.

This study presents a very simple and robust approach for preparing a pH-responsive microneedle (MN)-based drug-delivery system. We coated a porous polymer layer on stainless-steel (SS) microneedles characterized by pH-responsive formulation for transdermal drug delivery in response to pH changes. Figure 1 schematically illustrates this responsive release system and the corresponding mode of operation. The porous coatings were prepared by means of a dipping method, and NaHCO_3_ was encapsulated in the aqueous core of each pore. A thin polymer membrane was coated on this porous layer to be used as a “gatekeeper” to cap the pores of the porous layer in order to prevent the leakage of the pH-responsive formulation and drug, allowing the diffusion of protons inside the pores. The key component of this system is NaHCO_3_, which can be effortlessly incorporated into the pores of porous polymer coatings together with a drug, using an emulsion method. NaHCO_3_ is a gas-generating agent under acidic conditions. Reaction of NaHCO_3_ and an acid yields a salt and carbonic acid, which easily decomposes to carbon dioxide (CO_2_) and water (Figure 1) [22,23]. This effect generates pressure inside the pores of the porous polymer coating and ruptures the thin polymer membrane, thereby releasing the encapsulated drug. Skin pH is normally acidic, with a pH in the range of 4.2 to 5.6. The pH value of the epidermis is approximately 5.5. Skin cancer generally develops in this layer. This acidic environment can stimulate the porous polymer-coated MNs to release the encapsulated drug [21]. In addition, the existing pH of tumor tissue (mildly acidic) was considered an ideal trigger for the selective release of anticancer drugs from the developed MNs in tumor-targeted studies [24]. In many skin diseases where pH level is acidic like ichthyosis, this technology will be very useful for the targeted and control delivery of therapeutic agents [25]. Mammalian tissues are bathed in a milieu that typically contains ~25 mM HCO_3_^−^, and cells developed a means of taking up extracellular HCO_3_^−^ for alkalinization of their cytosol [26]. Additionally, NaHCO_3_ is used as a buffer agent in the cell culture medium. The efficiency of the pH-responsive porous polymer-coated MNs as a drug-delivery system was determined, and the release profiles of model drug molecules were assessed at stimulated pH of 7.4 and 5.5. Extremely small amounts of drug were released from MNs containing NaHCO_3_ at pH 7.4. This suggests that proton (H^+^)-ion diffusion through the thin poly(lactic-*co*-glycolic acid) (PLGA) membrane into the MN pores was prevented. The consequent reaction with sodium bicarbonate and the corresponding pressure due to carbon dioxide (CO_2_) creation inside the pores were also prevented. This resulted in almost no drug release from the porous polymer-coated MNs under normal physiological conditions. When the MNs were exposed to PBS at pH 5.5 (acidic condition), a significant amount of drug was released. Nearly 98% of the encapsulated drug was released in the test medium within 60 min. This confirmed the pH dependence of encapsulated drug release from the porous polymer-coated MNs with NaHCO_3_-containing pores.

## 2. Materials and Methods

### 2.1. Materials

All the chemicals for this study were purchased from Sigma-Aldrich and used without further purification. The following chemicals were employed: poly(lactic-*co*-glycolic acid) (PLGA, lactide:glycolide = 65:35, molecular weight (MW): 40,000–75,000), poly(vinyl alcohol) (PVA, MW: 31,000–50,000, 98–99 % hydrolyzed), dichloromethane (DCM, anhydrous, ≥99.8%, ρ: 1.325 g/mL, MW: 84.93 g/mol, melting point (mp): −97 °C), poly(methyl methacrylate) (PMMA, also known as acrylic plastic), polyethyleneimine (PEI, analytical standard, 50% (*w*/*v*) in water), calcein dye, lidocaine (MW: 234.34 Da), and phosphate-buffered saline (PBS). Stainless-steel MNs were obtained from UBioMed (Daegu, Korea) and sodium bicarbonate (NaHCO_3_) was bought from the local market (Daegu, Korea). 

### 2.2. Coating Solution Preparation and Porous MN Fabrication

MNs were fabricated via micromachining technologies. To fabricate the MN array, a 10-mm square plate of poly(methyl methacrylate) was machined to a height of 4.5 mm. A jig was used to fashion holes for 49 microneedles. The height and diameter of the MNs on the array were 0.60 mm and 0.12 mm, respectively. An array of 7 × 7 microneedles with intervals of 0.40 mm was prepared by inserting the needles into an acrylic plate. 

The polymer coating solution was prepared using a water in oil emulsion method [27]. Briefly, 1 mL of aqueous PVA (10 mg/mL) that contained NaHCO_3_ (50 mg/mL) and 0.25 mL of aqueous lidocaine solution (4 mg/mL) was homogenized with 4 mL of PLGA solution (20 mg/mL in DCM) for 3 min to form the coating solution. The microneedles were cleaned using acetone, deionized (DI) water, and ethanol and dried prior to the plasma treatment. After drying, the MNs were placed in a plasma chamber and subjected to an oxygen-plasma discharge for 5 min. The plasma generator operated at a frequency of 13.56 MHz (RF-GEN, IDT Eng. Co., Carson, NA, USA), and the specifications of the plasma discharge were as follows: plasma power (260 W), time (5 min), and oxygen pressure (0.2 mTorr). After the plasma treatment, the samples were removed from the plasma chamber and submerged in a solution of 2 % PEI (*w*/*w*) in water for 5 min. The samples were then submerged in DI water for 1 min (in triplicate) and subsequently immersed in the coating solution for 5 min to allow coating of the porous polymer layer on the MNs. After the formation of the porous polymer coating, a thin membrane on the porous coating was formed by dipping MNs in another PLGA solution (5 mg/mL).

Subsequently, for complete evaporation of the solvent, the samples were kept at room temperature for 2 h. 

### 2.3. Characterization and Parametric Study

The surface morphology and size of the as-prepared porous coatings on MNs were investigated via stereo microscopy and field-emission scanning electron microscopy (FE-SEM; HITACHI S-4800, Tokyo, Japan).

The drug loading efficiency (DLE) of lidocaine was tested using the ultraviolet (UV) spectrophotometry method [28]. An aliquot of 1.5 mL of the supernatant was measured for absorbance using UV spectrophotometry (Ocean Optics, USB 4000, Winter Park, FA, USA). The absorbance of lidocaine was recorded and the concentration of free lidocaine in the supernatant was calculated from a standard curve. The DLE was calculated using the following formula:

(1) $$Drug\;loading\;efficiency=\frac{\mathrm{total}\;\mathrm{amount}\;\mathrm{of}\;\mathrm{lidocaine}\;\mathrm{added}-\mathrm{free}\;\mathrm{lidocaine}}{\;\mathrm{total}\;\mathrm{amount}\;\mathrm{of}\;\mathrm{lidocaine}\;\mathrm{added}}\times 100\%.$$

Similarly, to inspect the effect of NaHCO_3_ on the lidocaine release, two samples were prepared. In the control sample (B = NaHCO_3_, L = lidocaine), the contents of NaHCO_3_, and lidocaine were maintained as used in the whole study. In another sample, i.e., 1/2B = (1/2B, L), the amount of NaHCO_3_ was reduced to half. The two samples were incubated in PBS with pH 7.4 for 60 min at 37 °C. For the next 60 min, samples were incubated in PBS with pH 5.5 at 37 °C. The cumulative release of lidocaine was calculated using UV absorbance and a standard curve.

### 2.4. In Vitro pH-Responsive Drug Release of Porous Coated MNs

The model drug/lidocaine release from the pH-responsive porous coated MNs was evaluated in vitro in phosphate-buffered saline (PBS) with different pH levels at 37 °C. To verify the pH-responsive capability of these MNs, two samples each of 25 MNs were immersed in vials that contained 1.5 mL of PBS at pH 7.4 and at pH 5.5, respectively. The vials were incubated in a water bath at 37 °C. At predetermined times, samples were collected from the supernatant fluid. The model drug/lidocaine released from the MNs was calculated from the UV absorbance and the standard curve, which was obtained by derivatizing the known concentrations of the drug/lidocaine. The concentration of the delivered drug was then plotted on the standard curve and the masses of the drug delivered via the porous-coated MNs were calculated. The experiments were repeated five times, and mean values were used for the analysis. Furthermore, the release percentage of lidocaine was then estimated by dividing the amount of drug released in the receiving medium by the total amount of lidocaine added.

### 2.5. Insertion Capability and Diffusion Kinetics of Calcein Dye from Porous Coated MNs in Porcine Skin

To evaluate the insertion capability and drug diffusion kinetics of pH-responsive porous coated MNs in skin, calcein-loaded porous coated MNs containing NaHCO_3_ were applied to porcine cadaver skin. Full-thickness fresh porcine skin pieces were sourced from a local abattoir and immediately stored at −20 °C. Prior to the experiments, skin pieces were thawed for 1 h at room temperature. The hair was carefully removed using hair clippers and the skin surfaces were cleaned carefully with 75% alcohol and air-dried for 10 min. The pieces were placed on a fixed platform and pinned down to maintain slight tension across the surface. PBS at pH 7.4 and 5.5 was injected into the skin from the back side to control the required pH of the skin. After this, the MNs were manually inserted into the skin and placed in an oven at 37 °C for 5 min and then removed. The morphology of the retrieved porous MNs was observed via SEM. 

### 2.6. Confocal Laser Scanning Microscope (CLSM)

To examine the release and dispersion of drug (calcein) from pH-responsive porous coated MNs after insertion, the skin samples were recorded using a confocal laser scanning microscope (CLSM; C2, Nikon Corporation, Seoul, Korea). After drug delivery by individual microneedles, porcine skin samples were cut to appropriate sizes and embedded in optimal cutting temperature (OCT) compounds for histological sectioning. Sectioning was performed with a microtome (RM2235, Leica, Seoul, Korea) tool. The frozen OCT skin specimens were sliced into 30-μm-thick sections to be observed using a CLSM. The insertion depth of MNs and the release of drug could be observed directly from the bright-field and fluorescence images.

## 3. Results and Discussion

### 3.1. Synthesis and Characterization of pH-Responsive Porous Coated MNs

In a previous study, we reported a simple method (i.e., dipping method) of fabricating a uniform porous polymer coating on SS microneedles with the aim of improving the delivery rate [29]. The important component of the system was a porogen/aqueous gelatin, which can be easily incorporated into polymer coatings by using an emulsion method to create pores in the polymer layer. The pore size of the porous coating can be adjusted by changing the porogen-to-polymer ratio, as well as the homogenization rate, which in turn controls the drug formulation. The thickness of the porous layer depends on the concentration of polymer solution and the number of coated layers. 

**Figure 2:**
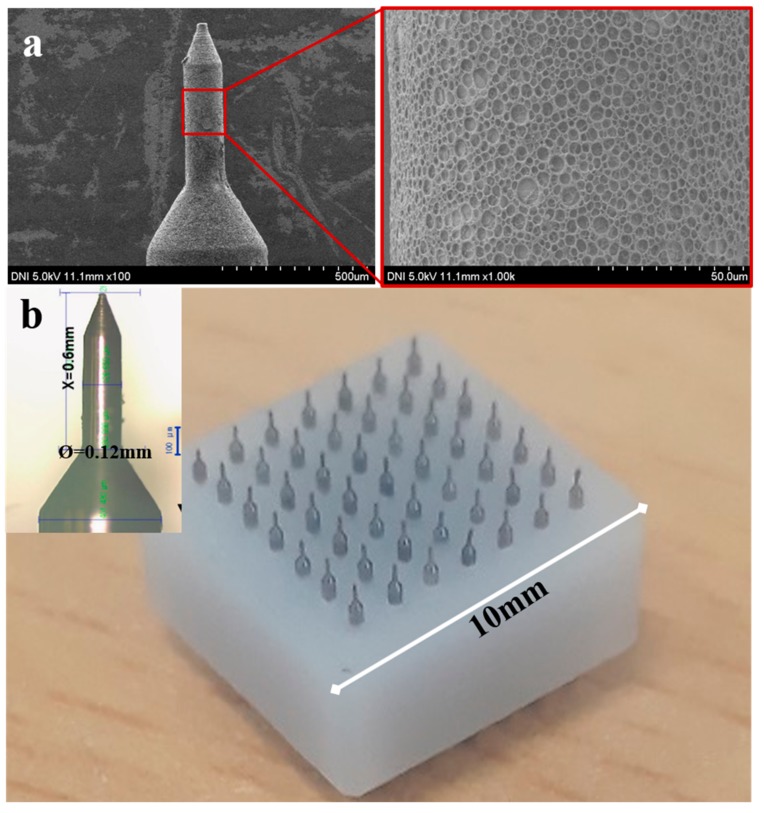
SEM images and Stereo micrograph of the pH-responsive porous coated microneedles (MNs): (**a**) SEM images at two different magnifications; (**b**) MN array (inset is magnified stereo micrograph of MN). The length and diameter of stainless-steel (SS) MN were 600 μm and 120 μm, respectively.

Herein, we fabricated a novel smart pH-responsive porous polymer coating on SS microneedles using the aforementioned dipping method. PLGA, a biodegradable and biocompatible polymer, was used as the coating polymer. Here, we used aqueous NaHCO_3_ as the porogen for incorporating pores into the polymer layer, and as a pH-responsive component in the porous polymer coatings. The porosity of the porous layer was affected by the ratio of aqueous NaHCO_3_ to polymer solution and the homogenization rate. As reported in our previous work, increasing the ratio and reducing the rate led to an increase in the pore size and vice versa [29]. Stereo microscopy and FE-SEM images were used to determine the porosity and uniformity of the pH-responsive polymer coating on the microneedles. All porous coated microneedles had essentially similar diameters within a narrow range of 140 μm to 146 μm (diameter of bare MN = 120 μm), demonstrating uniformity of the pH-responsive porous coated layer. Figure 2 shows a stereo micrograph and SEM images of the pH-responsive porous coated microneedles at various magnifications. The bright-field stereo micrograph revealed that all microneedles were uniformly coated with porous PLGA. The SEM images further confirmed that the porous coating was a uniform thin layer that had no effect on the needle architecture. Additionally, the polymer layer was highly porous with uniformly distributed pores on the microneedle peripheries. The results indicated good inter-connectivity between the pores, which provided pathways on the microneedle peripheries for the passage of drug formulations. 

### 3.2. In Vitro Drug Release of Porous Coated MNs in Response to pH Changes

**Figure 3:**
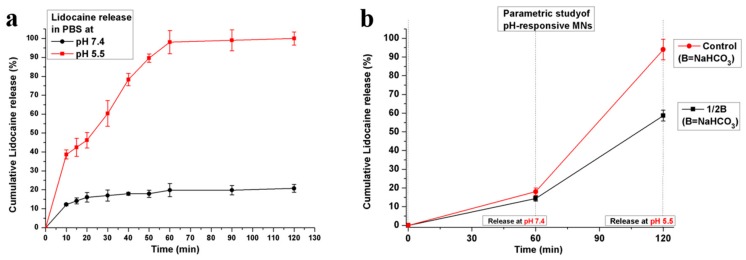
(**a**) Release profile of lidocaine for pH-responsive porous polymer-coated MNs incubated in a medium with different pH values that mimic a normal physiological environment (pH 7.4) and a mildly acidic environment (pH 5.5) at 37 °C; (**b**) release rate (line slope) of lidocaine as a function of NaHCO_3_ concentration in the release media for control (B, L) and 1/2B = (1/2B, L), containing one-half amount of NaHCO_3_ compared to control (B, L). Error bars indicate SD (*n* = 5).

To verify the effectiveness of the as-prepared porous polymer-coated MNs for pH-responsive drug-delivery applications, we evaluated the lidocaine release profiles of the MNs in phosphate-buffered saline (PBS). The MNs were incubated in a PBS medium with pH values of 7.4 and 5.5. For analysis, the medium was removed with a syringe at given time intervals. Figure 3a shows the profiles of lidocaine release from the MNs in PBS at 37 °C. An extremely small amount of lidocaine was released from the porous polymer-coated MNs with NaHCO_3_ (Porous MNs-w-NaHCO_3_) at pH 7.4. 

This suggests that the diffusion of proton (H^+^) ions through the PLGA membrane into the pores of the porous polymer-coated MNs was prevented. The corresponding reaction with sodium bicarbonate and, hence, the pressure due to carbon dioxide (CO_2_) creation inside the pores were also prevented. Accordingly, almost a negligible amount of drug was released from the MNs under normal physiological conditions. The small release of the drug in the initial 5 min may have resulted from the drug attached to the surface of MNs rather than the drug encapsulated inside the pores. For the next 2 h of incubation, the drug release increased negligibly, owing possibly to the very small pores in the PLGA membrane. Only a small amount of lidocaine was released in 2 h from porous polymer-coated MNs at pH 7.4. However, for MNs containing NaHCO_3_, a significant amount of drug was released when the MNs were exposed to PBS at pH 5.5 (mildly acidic environment). This confirmed the pH dependence of lidocaine release from MNs with NaHCO_3_-containing pores. Under these acidic conditions, the proton (H^+^) ions diffused through the thin membrane of PLGA into the pores of the MNs and reacted with NaHCO_3_. Accordingly, CO_2_ bubbles were generated inside the pores and pressure was produced. This effect created pores in the membrane or led to rupturing of the membrane, thus releasing the encapsulated lidocaine. Furthermore, when the amount of NaHCO_3_ was reduced to a half (1/2B = (1/2B, L)) vs. control (B, L) in the porous coated MNs, the rate of lidocaine release decreased correspondingly, as shown in Figure 3b. When both samples (control and 1/2B) of porous coated MNs were exposed to PBS with pH 7.4 at 37 °C, only a small amount of lidocaine was released in 60 min from both the samples. No significant difference was found in the release rate from both samples. However, when the samples were exposed to PBS with an acidic environment (pH 5.5) at 37 °C for the next 60 min, a significant amount of drug was released. However, the release rate of sample 1/2B compared to the control sample decreased correspondingly to the amount of NaHCO_3_ in the sample. Results showed that the content of NaHCO_3_ has great effect on the release rate of pH-responsive porous coated MNs. This implies the possibility of adjusting the drug release rate by varying the amount of NaHCO_3_ in porous coated MNs.

Collectively, the results substantiated that the release of model drug from the pH-responsive porous coated MNs undergoes a pH-mediated biomimetic process. This pH-responsive release behavior is of particular interest in relation to triggering the transdermal delivery of drugs.

**Figure 4:**
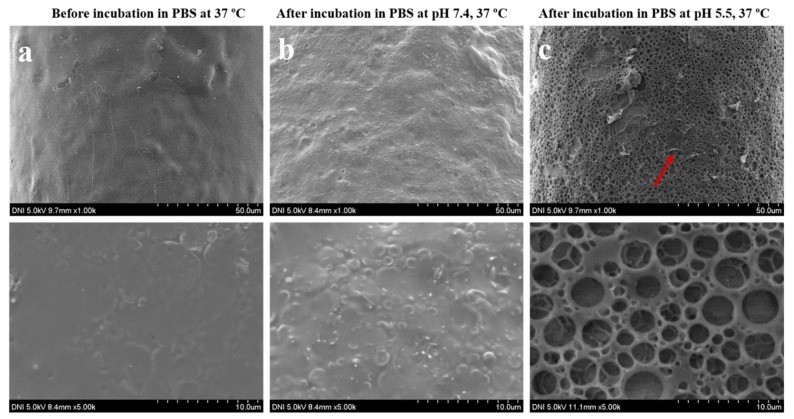
SEM images obtained at different magnifications of pH-responsive porous coated MNs: (**a**) before incubation; (**b**) after incubation at pH 7.4; (**c**) after incubation at pH 5.5 in phosphate-buffered saline (PBS) at 37 °C. The morphology of MNs before and after incubation at pH 7.4 was the same. The pores were unexposed, and the thin poly(lactic-*co*-glycolic acid) (PLGA) membrane stayed intact, whereas the pores of MNs incubated in PBS at pH 5.5 were open and the membrane was ruptured. The arrow indicates some traces of the remaining thin PLGA on the surface of the MNs.

To verify this hypothesis, FE-SEM images were obtained of the MNs before and after incubation in the acidic environment and under normal physiological conditions. The results showed that the surface morphology of pH-responsive porous polymer-coated MNs before and after incubation in PBS at pH 7.4 was the same. The thin PLGA membrane stayed intact, and the pores were unexposed to the medium. However, the membrane of the MNs was ruptured and the pores were open to the medium after incubating in PBS at pH 5.5, as shown in Figure 4. Approximately 60% of the encapsulated lidocaine was released in the test medium within the first 30 min, whereas nearly 98% was released in the subsequent 30 min. After the initial rupture of the membrane, most of the pores were exposed to the medium, and the encapsulated drug was released. However, some pores were only partially exposed to the medium during the first rupture of the membrane and, hence, a relatively long time was required for releasing the drug. After incubating in PBS at pH 5.5 for 2 h, almost all the lidocaine originally encapsulated within the pores of the MNs was released in the medium. The concentration of lidocaine, as determined via UV spectrometry, revealed this pH-sensitive release behavior of the as-prepared porous polymer-coated MNs. This pH-responsive release behavior is particularly relevant for triggering the transdermal delivery of drugs.

### 3.3. Insertion Capability and Diffusion Kinetics of Calcein Dye from MNs in Porcine Skin

**Figure 5:**
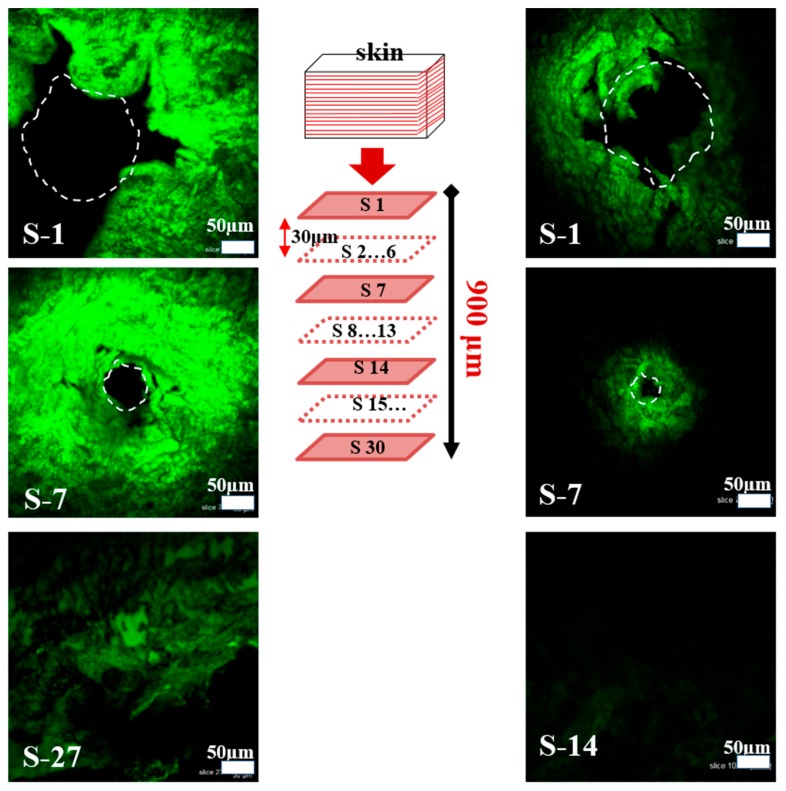
Confocal laser scanning micrographs of histological sections for calcein delivery in porcine skin at 37 °C: left side, calcein delivered in skin at pH 5.5; right side, calcein delivered in skin at pH 7.4. Histological sections were obtained by slicing the calcein-delivered skin tissues using a microtome. S-1 and S-27 represent the top skin section and the deepest section where calcein was detected, respectively. The thickness of each section is 30 μm (left side at pH 5.5). S-1 represents the top skin section, whereas S-14 represents the deepest section where calcein was detected (right side at pH 7.4).

The insertion ability and efficiency of drug delivery by the pH-responsive porous polymer-coated MNs in vitro were characterized. This was achieved by inserting MN arrays that contained calcein, a model drug, into porcine cadaver skin at 37 °C and monitoring the kinetics of its diffusion. We selected porcine skin rather than live animals for ethical reasons. In addition, previous studies showed that the delivery efficiency obtained in vitro in porcine skin is similar to that in vivo in mice [30,31]. The pH-responsive porous polymer-coated MNs were strong enough to be inserted into the skin without detaching and damaging the polymer coating, as reported in our previous work [29]. The MNs were inserted manually into the skin at pH 7.4 and 5.5 to assess their pH responsiveness. These pH values were maintained through subcutaneous injection (at 37 °C) of PBS at pH 7.4 and 5.5 into the skin. The length of SS MNs was 600 μm. Upon insertion, MNs penetrated to a depth of ~500 μm (Figure 5), as indicated by the resulting hole, which was observed only up to section 16 (S-16) (excluded from the figure). This difference in insertion depth may have resulted from deformation of the skin surface during MN insertion. Confocal laser scanning micrographs of histological sections obtained from the skin at pH 5.5 showed that the encapsulated calcein was successfully delivered into the relatively deeper tissues of the skin. Significant diffusion of the calcein occurred within 5 min. A strong green fluorescence was observed for the skin tissues from the MNs that penetrated the skin at pH 5.5. The distribution of calcein in tissues was further explored through CLSM images obtained at different depths of the skin (*z*-plane transverse sections). The depth of the pinholes in the skin (indicated by the white dotted lines) exceeded 480 µm, and the size of these pinholes decreased with increasing depth. Moreover, the diffusion of calcein was observed to a depth of 810 µm, as shown in Figure 5 (left side). On the left side of the figure, S-1 represents the top skin section of calcein delivery, whereas S-27 represents the lowest skin section where calcein was observed by means of CLSM images. The thickness of each section was 30 μm. However, for the skin at pH 7.4, most of the model drug (calcein) remained inside the pores of the pH-responsive porous polymer-coated MNs. A weak green fluorescence was observed around the area of the skin insert sites, in contrast to that observed under acidic conditions. Furthermore, calcein diffused only to the shallow tissues of the skin, and very weak fluorescence occurred at a depth of 420 µm. This small release of calcein under normal physiological conditions may be attributed to the calcein initially remaining on the surface of MNs during coating or a slight release through the very small pores of the thin PLGA membrane. As previously discussed, in the acidic skin environment, the NaHCO_3_ packed in the pores of the MNs reacted with the acid (protons), and diffused through the thin PLGA membrane. This generated CO_2_ bubbles and, in turn, pressure in the pores, leading to rupturing of the membrane and, hence, release of the encapsulated model drug (calcein). We also observed (from CLSM images) the diffusion areas and intensity of the fluorescence covered by the released model drugs at different depths of the skin. According to the results shown in Figure 5, the areas and intensity of the drug in the acidic environment were significantly greater than those associated with the normal physiological environment. The results obtained in vitro in porcine skin and in PBS were comparable. This revealed that our proposed porous polymer-coated MNs exhibit significant promise for use in smart pH-responsive drug-delivery applications.

## 4. Conclusions

In summary, we successfully developed a new biomimetic drug-delivery system using a pH-responsive formulation in porous polymer-coated MNs. Sodium bicarbonate, a pH-responsive component, was packed together with drugs in the pores of a porous polymer coating on SS microneedles. Once the pH-responsive porous polymer-coated MNs were exposed to an acidic microenvironment, protons diffused through the thin PLGA membrane into the pores of these MNs where they reacted with sodium bicarbonate (NaHCO_3_), thereby forming CO_2_. This effect generated pressure inside the pores of the coating and ruptured the membrane, leading to release of the encapsulated drug. Furthermore, FE-SEM images showed that, when the smart porous MNs were exposed to PBS at pH 7.4, the thin PLGA membrane stayed intact and the pores were closed. However, for MNs exposed to PBS at pH 5.5, the pores were opened, and the thin membrane burst. The in vitro studies demonstrated that the release of the drug from pH-responsive porous polymer-coated MNs was highly pH-dependent. Negligible release was observed for the MNs in receiving media at pH 7.4. However, significant drug release occurred when the MNs were exposed to acidic conditions (pH 5.5). Comparable results were obtained in vitro in porcine skin and in PBS. This revealed that our proposed stimuli-responsive porous MNs could potentially be used for the controlled release of drugs in an acidic microenvironment. Moreover, the stimuli-responsive drug carriers will enable on-demand controlled release profiles that may enhance therapeutic effectiveness and reduce systemic toxicity.
